# Exploring the clinically orientated roles of the general practice receptionist: a systematic review protocol

**DOI:** 10.1186/s13643-017-0612-6

**Published:** 2017-10-23

**Authors:** Michael Burrows, Nicola Gale, Sheila Greenfield, Ian Litchfield

**Affiliations:** 1 Institute of Applied Health Research, College of Medical and Dental Sciences, University of Birmingham, Edgbaston, Birmingham, B15 2TT UK; 2 School of Social Policy, HSMC Park House, University of Birmingham, Birmingham, UK

**Keywords:** GP receptionist, Primary care, Clinical roles, Triage

## Abstract

**Background:**

The receptionist is the focal point of the practice, undertaking an array of clinically orientated roles such as triaging patients for GP consultations or managing repeat prescribing. However, the full nature and extent of the receptionist’s clinical activities is unknown as are the implications for patients. The aim of the proposed review is to explore the nature of the receptionist’s clinical roles, their extent and their implications for patients. In doing so, we will highlight any gaps in the evidence base which future research may explore.

**Methods:**

The databases Medline/PubMed, Ovid, Cinahl, ASSIA, Cochrane, EMBASE and Science Direct will be searched for relevant literature. We will look at both qualitative and quantitative research on GP receptionists, based within primary care to explore their roles within the primary care team, the clinically relevant roles they undertake, the extent of these roles and any implications these roles might have. No limits are placed on the date or place of publication; however, only research published in English will be included. Screening, quality assessments and data extraction will be carried out by two reviewers, who are not blinded to study characteristics. Analysis follows a four-stage method, established by Whittemore and Knafl (2005).

**Discussion:**

The review will explore existing research covering the clinically orientated roles of the GP receptionist. The findings of the review will be important for healthcare professionals and academics working within primary healthcare. It will highlight and for the first time synthesise research relating to the complex and essential work of the GP receptionist. Our findings will inform the direction and focus of further research, as gaps in the knowledge base will be uncovered.

**Systematic review registration:**

PROSPERO registration no: CRD42016048957.

**Electronic supplementary material:**

The online version of this article (10.1186/s13643-017-0612-6) contains supplementary material, which is available to authorized users.

## Background

General practice receptionists are the first point of contact for patients. Their functions are varied and encompass administrative duties, such as filing, maintaining medical records and making appointments [1, 2]. In addition, the receptionist undertakes what can be described as clinically orientated roles which include repeat prescribing [2–4], interacting with patients [3, 5–11], making critical decisions and appointments (de facto triage) [5–7, 10, 11] and managing patients’ emotions [12]. The tasks they perform are rendered more difficult by working within an overtaxed primary care system [13]. The resulting pressure on primary care means that receptionists appear to be increasingly relied upon to assume clinically orientated roles. This affords medical staff more time to dedicate to their increasing clinical workload.

In a 2013 survey, 18% of GP practices surveyed reported that reception staff have some involvement in telephone triaging. Though the authors do not define ‘involvement’, it is clear this task has clinical implications as the inaccurate assessment of symptoms can lead to a delay in consultation and diagnosis or patients being referred to inappropriate services [10]. Though less visible, the receptionist’s role in repeat prescribing is considerable [4]. They bridge the gap between the request and the information held on file, using their own judgement to ensure and bolster patient safety and relying on the GPs to check accuracy [3].

Receptionists also report clinical information to patients, for example, the reporting of blood test results. In this scenario, receptionists relay results, sometimes with the help of pre-prepared scripts. Frequently, they are unable to respond to further enquiries from patients leading some to question whether this is an appropriate role for the receptionist [14]. Test result data has potentially serious clinical implications and the inability to provide surrounding information about a result can lead to anxiety in patients and discomfort for receptionists unable to offer support [14, 15].

Despite the range of clinically orientated duties, no formal training is required or systematically delivered. Instead, receptionists new in post typically receive their training from existing reception staff for training [2, 16]. Furthermore, GP surgeries are independent organisations and so budgetary and time constraints may mean that training for the receptionist is overlooked in favour of training for medical staff. The lack of any formal training can lead to issues around patient safety and care, including errors in directing patients to the correct service [10] or misinforming or poorly informing patients [14, 17]. These in turn may put the patient at risk and open the practice to severe penalties.

## Rationale

The receptionist is an essential feature of the primary care system, contributing to its smooth running and acting as point of contact and buffer, between patient, GP and other clinical staff. Primary care is under increasing pressure; the emphasis has shifted to prevention [13] and management of chronic conditions, and the needs of patients and the care available to them has become more complex. Within this environment, the receptionist is expected to fulfil a number of tasks with clear clinical implications yet without structured training or support; therefore, an up-to-date overview of this role is needed.

An earlier scoping review (undertaken by the lead author MB) indicates that the receptionist is female, married and undertakes a visible role in the practice as well as a number of clinical relevant tasks. However, a more systematic, concentrated search of existing literature is needed to fully explore these issues, to access the extent of the receptionist’s clinical roles and to identify if possible what implications the receptionist undertaking these roles might have.

The existing research is both qualitative and quantitative in nature and so our systematic review will be integrative. This will enable us to more fully explore all of the existing research and develop a comprehensive overview [18].

## Objectives

This review will summarise past research, draw conclusions and highlight unresolved issues and direct future research, in accordance with Whittemore and Knafl (2005). To do this, the review will explore the receptionist’s roles within the primary care team. It will also explore the literature around the clinically orientated roles of the GP receptionist, to identify the type and extent of the roles that these individuals undertake. The review will ask three questions:

1. What is the role of the GP receptionist within the primary care team?
2. What clinically orientated roles does the GP receptionist undertake?
3. What is the extent of these clinically orientated roles and the effects on the patient and patient care?

## Methods

The protocol has been prepared in line with the Systematic Review and Meta-analysis Protocols or PRISMA-P [19]. The PRISMA-P flowchart is provided in Additional file 1. In addition, the protocol has been registered with PROSPERO (CRD42016048957); the entry can be accessed via the following:

http://www.crd.york.ac.uk/PROSPERO/display_record.asp?ID=CRD42016048957

### Methodology

The systematic review will explore existing literature according to best practice in systematic review methodology [18]. The initial scoping review indicated a paucity of research in the field and what exists uses a number of methodological approaches. As such, the review will include both qualitative and quantitative research and provide a more comprehensive investigation of the topic.

### Eligibility criteria

The eligibility criteria used in the study are in accordance with the SPIDER search strategy [20]. SPIDER is a qualitative and mixed methods alternative search strategy to the more typical PICO tool [21] and stands for Sample, Phenomenon of interest, Design, Evaluation, Research type.

#### Sample

The research will include GP receptionists or medical secretaries, these are individuals tasked with providing administrative support to the practice (for example managing patient records, scanning documents or processing repeat prescriptions) and support for patients seeking medical care (booking appointments, dealing with patients attending the practice). In addition, they should be based in general practice, within primary care. This is operationalised as general practice based in the community offering care for minor or chronic/long term illness and providing referrals to specific services for more serious illness care.

#### Phenomenon of interest

The research included will cover the clinical and general roles of the receptionist as well as any potential implications for or effects on patients.

#### Design

The research will include interviews, focus groups, ethnographic observations, case series and surveys.

#### Evaluation

The review will explore attitudes, beliefs, satisfaction and medical outcomes.

#### Research type

Including qualitative, quantitative and mixed methodologies, only empirical research will be included. No limits are placed on the date of publication.

Research will not be excluded by country of origin. Though there may be differences in the structure, funding and support of medical systems around the world, research conducted within other healthcare models can still provide valuable information on the contrasting roles of the GP receptionist. However, research will only be included from outside the UK, where it is produced in English or a good quality translation is available.

### Search strategies

We will use multiple search strategies to obtain a representative sample [22]. Search strategies will be modified between databases where appropriate. The databases we will search are Medline/PubMed, Ovid, Cinahl, ASSIA, Cochrane, EMBASE and Science Direct. The search strategy will employ terms and alternatives to effectively capture all relevant research for the review, without a time limit. For example, the GP receptionist can be described as such or as a practice secretary or medical secretary.

See Additional file 2—for a detailed Medline search strategy.

Individual journals will be hand searched for relevant articles; these will include but not be limited to journals covering primary care research and healthcare research such as the British Journal of General Practice, The British Medical Journal (Open), The Journal for Health Care Quality and BMJ Quality and Safety, in addition to hand searching of the reference lists of included research. Finally, the review will include conference proceedings and National Health Service (NHS) or healthcare policy documents sourced from websites published by the NHS.

### Screening

#### Title and abstract screening

Literature searching will be undertaken by the lead author (MB) and search results will be extracted to Endnote X7.3.1 [23]. After this, stage duplicates will be removed by Endnote and MB will undertake the process of screening by title and abstract. Title screening will involve MB checking each title against the inclusion criteria. Studies deemed suitable for inclusion at this stage will be subjected to abstract review by the lead author; again, those meeting the inclusion criteria will be included for full-paper review.

#### Full-paper review

Two reviewers will undertake the full-text review. Microsoft Excel will be employed to facilitate this process. Quality assessment will be undertaken on each of the remaining papers; quality will be assessed using the CASP resource for qualitative research [24], the Quality Assessment Tool for Quantitative Studies [25] and the Mixed Methods Assessment Tool (MMAT) [26]. Studies will not be de facto excluded based on poor quality alone. Instead, the two reviewers will decide on exclusion based on the importance and worth of the research to the review, as well as overall quality. Each reviewer will independently review each article and will meet to discuss the inclusion or exclusion of papers at this stage. Where there is agreement between the reviewers, those papers will be included. However, where there are disagreements, these will be discussed and in the case of persisting disagreement, a third reviewer will be consulted.

#### Data management

As discussed, the reviewers will employ EndNote X7.3.1 [23] to facilitate the title and abstract review and Microsoft Excel will be used for full paper screening and quality assessment.

**Fig. 1:**
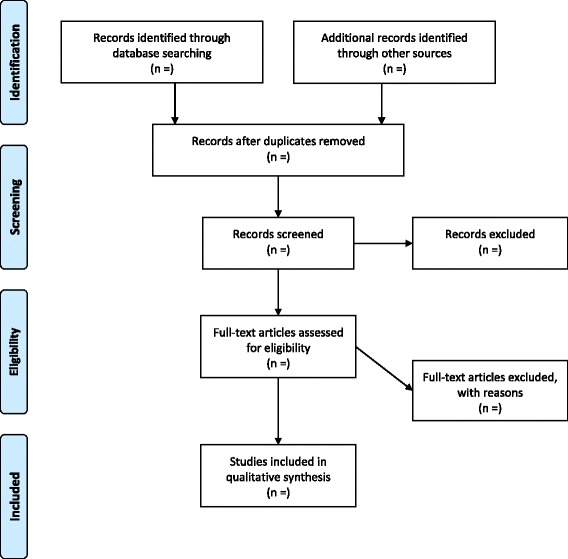
PRISMA flow diagram

Finally, a PRISMA diagram [19] will be created to show the flow of studies through the various levels of assessment (see Fig. 1).

### Data extraction

Data will be extracted independently by the lead author (MB) and a second reviewer. Data extraction will be based on the supplementary guidance chapter 5 to the Cochrane handbook [27]. This template will be modified to extract all data relevant to the research questions. Discrepancies in data extraction and entry will be resolved in discussion with a member of the wider research team. The reviewers will not be blinded to study characteristics. Data extracted will cover information relevant to the review questions established and include:

1. Publication information (author, contact details, funding sources, date of publication),
2. Study characteristics (research setting, design, method),
3. Participant information (number of participants, demographic information, including the age, sex/gender, marital status, educational attainment and additional protected characteristics, inclusion/exclusion criteria),
4. Outcomes (clinically orientated roles, time taken in these roles, developments in the role of the receptionist, potential effects of these clinical roles).

### Data analysis

Data analysis will follow the four-stage procedure detailed by Whittemore and Knafl (2005):

(i) Data reduction

Analysis begins with the process of data reduction. Included studies will be divided into groups based on the research design; as such, there will likely be four subcategories:

1. Qualitative research: including interviews, focus groups and ethnographic observations.
2. Case series
3. Surveys (surveys with open responses will be included and analysed with the qualitative research)
4. Mixed methods (i.e. consisting of a qualitative and quantitative element)

This process provides a systematic framework for the analysis.

Data reduction continues with each primary source being coded, employing a priori codes relevant to the review questions (for example, coding for clinical activities, such as repeat prescribing). From each of the studies included, a master file is created. This master file contains all of the codes derived from each study and is the basis for the continued analysis, enabling easier and more systematic comparison and integration of data on specific issues such as sample characteristics, surgery type, receptionist workload/roles or other variables.

(ii) Data display

The individual master files for each of the selected articles will be combined into a single Excel database for each subcategory defined in the reduction stage. At this stage, the process of reconstructing the data begins and within each of the sub categories, the individual codes are drawn together and grouped by similarity. For example, codes showing a clinical aspect can be grouped together as ‘clinical roles’ at this stage. The process of data display facilitates the recognition of patterns and relationships in the data and enables the development of early broad codes. These will in turn inform the direction of the analysis and the emerging themes.

(iii) Data comparison

This iterative stage of the analysis employs the data displays to compare the selected articles across all of the categories and subcategories. This allows for the identification of patterns, themes and relationships that may be present in the data to be uncovered and for the emerging core themes to be realised and then saturated. For example, a number of studies may discuss a number of clinically relevant duties that the receptionist has, each of these could then be coded across the whole dataset and result in codes for each of the different clinical activities, triage, clinical information provision and repeat prescribing, for example. NVivo v11 [28] will be employed, as it allows the user to examine similarities between the codes and the sources which inform those codes. This will enable a more robust integration of the codes.

(iv) Conclusion drawing and verification

The codes developed across the dataset will be further integrated with each other to form higher level descriptive codes/themes. As in the example given, each of the codes relating to triage, clinical information provision and repeat prescribing are conceptually related as different aspects of clinical work and so could be integrated into a single theme.

## Discussion

This review will produce a comprehensive account of the existing literature covering the clinically orientated roles of the GP receptionist. The review’s findings will highlight the limitations and gaps in the existing literature and will in turn inform the authors’ ongoing research [29] which is funded by the Health Foundation [project reference—7452].

The findings of this review will also be important for healthcare professionals and academics working within the primary healthcare field. The review aims to clarify the roles that the receptionist undertakes, issues around training and any potential implications of the receptionist taking on clinically orientated roles. Furthermore it will highlight the potentially problematic ad hoc adoption of clinically orientated roles by untrained staff or on the contrary highlight this as a continuation of roles that the receptionist has been undertaking for decades.

The review will explore the need for any additional training for the GP receptionist. It will raise awareness of the need for closer attention to the roles they undertake and the support that is available in practice for this. The GP 5-year forward view details 45 million pounds of funding for the training of receptionists to undertake two discrete roles: managing clinical correspondence and active signposting (care navigating) [30]. There is scope for the role of receptionists to be expanded to take on more medical roles such as phlebotomy or taking blood pressure. This is especially interesting given the move of general practice towards multi-disciplinary team working and the potential this provides for expanding the role of receptionists [31].

Finally, the review will highlight the importance of the receptionist as a focal point of general practice, a role that has been potentially overlooked by the research community and in practice.

## Additional files

Additional file 1: PRISMA-P 2015 checklist. (DOCX 36 kb)

Additional file 2: Medline search strategy. (DOCX 15 kb)

## Abbreviations

ASSIA: Applied Social Sciences Index and Abstracts
BMJ: British Medical Journal
CASP: Critical Appraisal Skills Programme
CINAHL: Cumulative Index to Nursing and Allied Health Literature
GP: General practice
GPs: General practitioners
MMAT: Mixed Methods Appraisal Tool
NHS: National Health Service
PRISMA: Preferred Reporting Items for Systematic Reviews and Meta-Analyses
PRISMA-P: Preferred Reporting Items for Systematic Reviews and Meta-Analyses Protocol
SPIDER: Sample, Phenomenon of Interest, Design, Evaluation, Research type
UK: United Kingdom
