# Supplementary material for: Exploring the clinically orientated roles of the general practice receptionist: a systematic review protocol
Source: Syst Rev. 2017 Oct 23;6:209. doi: 10.1186/s13643-017-0612-6 (PMC5651636; doi:10.1186/s13643-017-0612-6)
Supplement: Supplementary file 2 — Medline search strategy. (DOCX 15 kb) [file 13643_2017_612_MOESM2_ESM.docx]

**Additional File 2: Medline Search Strategy**

| 1 | ‘GP receptionist$’ |
| --- | --- |
| 2 | ‘General practice receptionist$’ |
| 3 | ‘Practice receptionist$’ |
| 4 | ‘Receptionist$’ |
| 5 | ‘Role$’ |
| 6 | ‘Clinical role$’ |
| 7 | ‘Clinical work’ |
| 8 | ‘Clinical function$’ |
| 9 | ‘Medical role$’ |
| 10 | ‘Medical function$’ |
| 11 | ‘Job satisfaction’ |
| 12 | ‘Attitudes’ |
| 13 | (1 or 2 or 3 or 4) and (5 or 6 or 7 or 8 or 9 or 10 or 11 or 12) |
| 14 | ‘Patient outcome$’ |
| 15 | ‘Patient satisfaction$’ |
| 16 | ‘Patient participation’ |
| 17 | ‘Patient effects’ |
| 18 | ‘Patient view$’ |
| 19 | ‘Patient attitude$’ |
| 20 | 13 and (14 or 15 or 16 or 17 or 18 or 19) |
| 21 | ‘Primary care’ |
| 22 | ‘Primary healthcare’ |
| 23 | ‘GP practice’ |
| 24 | ‘General practice’ |
| 25 | ‘GP Surger$’ |
| 26 | ‘General practice management’ |
| 27 | ‘General practice staff’ |
| 28 | 20 and (21 or 22 or 23 or 24 or 25 or 26 or 27) |
| 29 | (1 or 2 or 3 or 4) adj2 (14 or 15 or 16 or 17 or 18 or 19) |
| 30 | (1 or 2 or 3 or 4) adj2 (5 or 6 or 7 or 8) |
